# Fibroblast activation protein inhibitor radiotheranostics for imaging activated tumor stroma and guiding radionuclide therapy

**DOI:** 10.3389/fonc.2026.1895276

**Published:** 2026-07-10

**Authors:** Hangyu Wu, Ze Liu

**Affiliations:** 1 Department of Radiology, Ningbo Hangzhou Bay Hospital (Ningbo Branch of Renji Hospital, Shanghai Jiao Tong University School of Medicine), Ningbo, Zhejiang, China; 2 Department of Nuclear Medicine, Ningbo Hangzhou Bay Hospital (Ningbo Branch of Renji Hospital, Shanghai Jiao Tong University School of Medicine), Ningbo, Zhejiang, China

**Keywords:** cancer-associated fibroblasts, FAP inhibitor, fibroblast activation protein, fibro-inflammatory uptake, PET/CT, radioligand therapy, radiotheranostics, tumor microenvironment

## Abstract

Fibroblast activation protein (FAP) is expressed by activated fibroblasts and selected vascular-associated stromal cells in tumors and non-malignant repair processes. Radiolabeled FAP inhibitors (FAPIs) allow PET mapping of FAP-accessible activated stroma, complementing tumor-cell metabolic imaging with FDG. This narrative review summarizes the biological basis of FAP targeting, radiopharmaceutical design, clinical imaging applications, benign fibro-inflammatory pitfalls, and early therapeutic development. FAPI PET can provide high lesion-to-background contrast in desmoplastic and low-FDG-avid tumors and in regions where physiologic FDG uptake complicates interpretation. The strongest current clinical scenarios include problem-solving in gastrointestinal cancers, peritoneal disease, pancreatic ductal adenocarcinoma, hepatocellular carcinoma, and biliary tract cancers, as well as whole-body assessment of target expression before therapy trials. However, FAPI uptake is not tumor specific; wound healing, post-treatment remodeling, fibrosis, arthritis, pancreatitis, liver disease, atherosclerosis, and other fibro-inflammatory or repair states require careful correlation with CT, MRI, FDG PET, clinical history, and follow-up. For therapy, high early PET contrast alone is insufficient. First-generation monomeric tracers may wash out rapidly, FAP expression is heterogeneous, and absorbed dose depends on ligand residence time, lesion architecture, perfusion, radionuclide range, normal-organ uptake, marrow reserve, and the fraction of disease that is FAP avid. Longer-retention ligands, dimeric or multimeric constructs, albumin-binding approaches, and beta- or alpha-emitter strategies are expanding clinical development, but prospective dosimetry and outcome data remain limited. Overall, current evidence supports FAPI PET as a complementary imaging method and selection tool for protocolized FAP-targeted radionuclide therapy rather than as a universal replacement for established imaging or a routine therapy platform. Future translation should emphasize standardized acquisition and reporting, disease-specific indications, benign uptake checkpoints, lesion-level dosimetry, physiologically relevant preclinical models, and prospective trials measuring management impact, toxicity, quality of life, and patient-relevant outcomes.

## 1 Introduction

Precision oncology increasingly uses whole-body biomarkers to describe not only malignant cells but also the tissue environment in which tumors grow. The tumor microenvironment (TME) includes fibroblasts, immune cells, endothelium, extracellular matrix (ECM), cytokine networks, and metabolic gradients. Among these components, cancer-associated fibroblasts (CAFs) are clinically relevant because they participate in matrix remodeling, invasion, angiogenesis, immune exclusion, and resistance to therapy (1).

Fibroblast activation protein is a type II transmembrane serine protease with dipeptidyl peptidase and endopeptidase activity. It is usually low in most quiescent adult tissues but is upregulated in wound healing, fibrosis, chronic tissue injury, and the stroma of many epithelial and mesenchymal malignancies (2, 3). In this review, the term “fibro-inflammatory” is used deliberately to describe inflammatory settings in which the PET signal is expected to arise from activated fibroblasts, myofibroblasts, pericyte-like cells, or vascular smooth-muscle-cell-related stromal cells, rather than from inflammatory leukocytes themselves. This wording is clinically important because FAPI uptake often accompanies inflammation, but the cellular source is typically activated mesenchymal stroma stimulated by inflammatory injury (4, 5).

The clinical FAP imaging field was accelerated by quinoline-based inhibitors that can be labeled with diagnostic or therapeutic radionuclides. Early 68Ga-labeled tracers demonstrated rapid blood-pool clearance, low background activity in several normal organs, and conspicuous uptake in many tumor types (6–11). Subsequent work extended the platform to 18F-labeled and kit-based agents, improved production logistics, and therapy-oriented ligands (12, 13).

This narrative review evaluates FAPI-based radiotheranostics for precision oncology. The emphasis is on clinically actionable mechanisms: why stromal imaging differs from FDG PET, how ligand design affects diagnostic and therapeutic performance, which disease settings are most plausible, why benign uptake must be reported carefully, and what evidence is still needed before FAP-targeted radionuclide therapy can move beyond early clinical development.

## 2 Biological rationale: mapping FAP-accessible activated stroma

FAP-targeted imaging differs from many tumor-cell-directed approaches because the visible signal largely reflects activated stromal biology. This distinction can be useful when tumor-cell metabolism is low, heterogeneous, or obscured by physiologic tracer activity. In contrast, activated fibroblast-rich stroma may outline invasive margins, peritoneal implants, desmoplastic reaction, and metastatic niches that are not well characterized by morphology alone (7–9).

CAFs are not a single cell type. Myofibroblastic, inflammatory, antigen-presenting, and vascular-associated CAF states can coexist within one tumor, and their distribution changes with hypoxia, therapy, and local tissue injury. FAP expression therefore samples only one compartment of stromal biology, and a FAPI PET scan should be interpreted as an image of FAP-accessible activated mesenchymal cells rather than a direct map of tumor-cell number. This is also why the association between FAP signal and immune phenotype is biologically plausible but not interchangeable with immune-cell infiltration; high FAP expression in colorectal cancer, for example, has been associated with angiogenesis and immunoregulation processes (14).

The biology has different implications for diagnosis and therapy. For diagnosis, stromal uptake can help detect lesions with low FDG avidity or lesions located near organs with high physiologic FDG activity. For therapy, a stromal target may expose tumor-supporting fibroblasts and nearby tumor cells to radiation by crossfire, but treatment effectiveness depends on ligand residence time, radionuclide path length, lesion size, perfusion, tissue penetration, internalization or membrane retention, and the fraction of disease that is FAP avid. Thus, high diagnostic contrast is necessary but not sufficient for effective FAP-RLT (15–26).

## 3 Radiopharmaceutical design and pharmacokinetic considerations

### 3.1 Diagnostic tracers

The first widely used FAPI tracers combine a FAP-binding inhibitor with a linker and a chelator such as DOTA. The inhibitor drives binding, whereas linker charge, hydrophilicity, chelator geometry, and radiometal selection influence renal clearance, nonspecific uptake, tumor retention, and image timing (6, 10, 11). For diagnostic PET, rapid background clearance is often advantageous because high contrast can be achieved soon after injection.

68Ga labeling enabled broad clinical testing because generator-produced 68Ga can be used in centers without a cyclotron, but generator yield limits batch size and distribution. 18F-labeled tracers may support larger batches, longer distribution, and multicenter studies, but each tracer still requires validation of synthesis, stability, uptake time, biodistribution, dosimetry, and quantitative harmonization (12, 13). The 2025 SNMMI/EANM FAP PET procedure standard is important because multicenter evidence depends on comparable production, acquisition, reconstruction, and reporting (12).

### 3.2 Therapy-oriented ligand optimization

Therapy imposes stricter pharmacokinetic requirements than imaging. A tracer that clears quickly can be excellent for diagnostic contrast but insufficient for radiation delivery. FAP-targeted therapy requires enough residence time in tumor and an acceptable tumor-to-critical-organ absorbed-dose ratio. Several strategies are being investigated: increasing avidity by dimerization or multimerization, extending circulation through albumin binders or fatty-acid conjugation, using cyclic peptides or boronic acid motifs with different binding/internalization behavior, and modifying linkers or chelators to alter renal and hepatic handling (15–23).

Monomeric quinoline-based FAPI agents such as FAPI-04 and FAPI-46 are attractive diagnostic tracers because of rapid clearance and high early contrast. Their limitation for therapy is short residence time in many tumors. Dimeric and tetrameric constructs can increase apparent avidity and reduce off-rate, and preclinical and early clinical studies suggest higher tumor retention in selected designs (16–19). The trade-off is that greater size and multivalency may change renal uptake, hepatobiliary clearance, nonspecific binding, and manufacturability; therefore a dimer should not be assumed to be safer or more effective without matched dosimetry.

Albumin-binder and fatty-acid strategies address washout by prolonging circulation and repeated tumor exposure (20, 21). This can improve time-integrated tumor activity, but the same prolonged blood residence may increase red-marrow exposure and background activity. The clinically relevant endpoint is not absolute tumor SUV or tumor half-life alone, but therapeutic index: tumor absorbed dose divided by dose to marrow, kidneys, liver, bowel, salivary glands, or other dose-limiting tissues for the specific compound. Prolonged circulation is therefore beneficial only when it increases this ratio rather than simply increasing whole-body dose.

Emerging long-retention FAP ligands illustrate this balance. FAP-2286, a cyclic peptide with DOTA, showed substantially longer tumor retention than FAPI-46 in FAP-expressing xenografts and subsequently entered clinical development (22). PNT6555, a boronic acid-based FAP-targeted radiotheranostic, was developed with attention to cellular uptake/internalization and therapeutic radionuclide compatibility (23). These examples support a shift from “detectable target” to “deliverable absorbed dose” as the central criterion for therapy ligand selection.

Preclinical model choice is a critical but often underemphasized determinant of apparent efficacy. Many early studies used cancer cell lines engineered to overexpress human FAP, which are useful for controlled binding experiments but may not reproduce the density, spatial distribution, stromal localization, and vascular accessibility of human CAF-associated FAP. Comparative evaluation of transduced and endogenous/preferentially stromal FAP models shows that model selection can change tracer binding and *in vivo* interpretation (24). Therapy-oriented development should therefore prioritize models with physiologically relevant FAP expression, including stromal or orthotopic models when feasible, and should report FAP density, stromal localization, perfusion, and normal-organ uptake alongside tumor growth delay.

### 3.3 Radionuclide, chelator, and internalization considerations

Chelator selection determines which radiometals can be used and how stable the final complex is under labeling and *in vivo* conditions. DOTA-containing agents are attractive because they can accommodate several diagnostic and therapeutic radiometals, but labeling temperature, molar activity, metal contaminants, and complex stability remain agent-specific variables. Theranostic pairing should therefore be based on matched biology and measured pharmacokinetics rather than on the chelator alone (6, 10, 22, 23).

For therapy, beta emitters such as 177Lu or 90Y and alpha emitters such as 225Ac or 213Bi have different advantages. Beta particles may be better suited to heterogeneous or larger lesions because of longer path length and crossfire, whereas alpha particles offer high linear energy transfer over a short range. The optimal pairing depends on lesion size, FAP distribution, internalization or membrane retention, normal-organ dose, and whether the clinical goal is disease stabilization, symptom palliation, local control, or cytoreduction (25, 26).

Internalization deserves specific mention. For residualizing radiometals, internalization can increase cellular retention and absorbed dose; however, FAP is a stromal surface target and clinically useful activity may also arise from persistent membrane binding and crossfire from beta emitters. The importance of internalization is therefore ligand- and radionuclide-specific. Agents designed to improve cellular uptake or durable binding should be compared not only by *in vitro* internalization percentages, but also by serial tumor dosimetry and tumor-to-marrow and tumor-to-kidney ratios in relevant models (23). Representative FAPI radiopharmaceutical design strategies and their translational implications are summarized in **Table 1**.

**Table 1:** FAP inhibitor radiopharmaceutical design strategies and translational implications.

Strategy/examples	What it improves	Main trade-off
Monomeric quinoline FAPI (FAPI-04, FAPI-46) (6–11)	Rapid background clearance and high diagnostic contrast.	Tumor residence may be too short for effective radionuclide therapy.
18F labeling or kit-based 68Ga agents (e.g., FAPI-74) (12, 13)	Larger batches, distribution, and multicenter standardization.	Each tracer still requires agent-specific validation.
Dimeric or multimeric FAPI (16–19)	Higher avidity and longer retention in selected designs.	May increase kidney uptake, blood-pool activity, or nonspecific binding.
Albumin-binding or fatty-acid conjugates (EB-FAPI/LNC1004 class) (20, 21, 50, 51)	Higher time-integrated tumor activity for therapy.	Prolonged circulation can increase marrow or blood dose.
Radionuclide and chelator pairing (177Lu, 90Y, 225Ac, 213Bi) (19, 25, 26, 45–52)	Matches particle range and dose rate to lesion size and heterogeneity.	Requires serial dosimetry and toxicity monitoring.
Preclinical model selection (24)	Physiologic FAP-expression models improve translational relevance.	Artificial FAP overexpression can overestimate therapeutic index.

## 4 Clinical imaging applications of FAPI PET

### 4.1 Relation to FDG PET

FAPI PET should be framed as complementary to FDG PET rather than as a universal replacement. FDG PET is deeply validated in many cancers and reflects glucose metabolism, treatment inflammation, and viable tumor burden in ways that FAP imaging does not. FAPI PET adds value when stromal activation is the more informative signal or when FDG interpretation is limited by low tumor avidity or high physiologic background (27–32).

Head-to-head studies and systematic reviews show that FAPI PET often has high lesion-to-background contrast in many tumor types, but the degree of benefit is disease-specific. The most persuasive uses are those tied to a defined clinical question: detecting occult peritoneal disease, refining local extent for radiotherapy planning in prospective settings, clarifying equivocal lesions, assessing whole-body FAP expression before therapy trials, or identifying discordant biology across metastases (30–32).

For everyday interpretation, discordance between FAPI and FDG should be described rather than treated as an error. FDG-positive/FAPI-negative lesions may represent tumor-cell-dominant disease, metabolically active inflammation, or FAP-negative progression; FAPI-positive/FDG-negative lesions may reflect stromal-rich tumor or benign repair. Multidisciplinary correlation is essential whenever FAPI PET is used to alter a surgical plan, radiation volume, or systemic treatment strategy (30–32).

### 4.2 Gastrointestinal, pancreatic, liver, breast, thoracic, and other tumors

Gastrointestinal cancers remain among the strongest clinical settings for FAPI PET. Diffuse or signet-ring gastric cancer can have variable FDG avidity, and peritoneal implants can be difficult to detect on CT. A systematic review of gastric cancer and early peritoneal carcinomatosis reports support FAPI PET as an adjunct for staging and recurrence assessment, particularly when management depends on whether disease is localized or disseminated (33, 34).

Liver malignancies merit explicit attention. FDG PET has limited sensitivity for well-differentiated hepatocellular carcinoma (HCC) and can be compromised by high physiologic hepatic background. In a retrospective HCC comparison, 68Ga-FAPI-04 PET/CT showed higher sensitivity than FDG PET/CT for intrahepatic lesions (85.7% versus 57.1%), including small tumors of 2 cm or less (68.8% versus 18.8%) and well- or moderately differentiated tumors (83.3% versus 33.3%) (35). These data support FAPI PET as a complementary liver imaging biomarker, while recognizing that cirrhosis, fibrosis, inflammation, and post-locoregional therapy can produce fibro-inflammatory uptake.

Biliary tract cancer and cholangiocarcinoma are also biologically plausible because they are frequently desmoplastic. In a prospective biliary tract cancer study, 68Ga-FAPI PET/CT showed higher sensitivity than FDG PET/CT for primary tumors, nodal metastases, and distant metastases, and altered stage or restage assignment in a subset of patients (36). Additional cholangiocarcinoma experience with FAPI-46 suggests improved tumor detection compared with FDG PET/CT and CT in selected patients (37). These findings justify greater attention to HCC and biliary tract cancer in disease-specific trials, but they should not be generalized to all liver lesions without morphologic and clinical correlation. In lower gastrointestinal malignancies, early clinical experience suggests that FAPI PET can improve delineation of primary and metastatic disease in selected patients (38). Pancreatic ductal adenocarcinoma is biologically attractive because of its dense desmoplastic stroma and difficult local assessment; early 18F-FAPI-74 data support further evaluation for staging and lesion detection, while radiation target definition remains an investigational application requiring disease-specific planning validation (39).

Breast cancer, lung cancer, sarcoma, and glioblastoma illustrate the breadth and limitations of FAP imaging. FAPI PET/MRI has depicted breast tumors and nodal disease with low background in some anatomic regions (40). FAPI-74 has been evaluated for biodistribution, dosimetry, and tumor delineation in lung cancer (13). In glioblastoma, correlations with perfusion or diffusion imaging are incomplete, supporting the concept that FAPI PET measures a distinct stromal or vascular-associated feature rather than conventional cellularity (41).

These examples show why disease-specific validation matters. A scan that clarifies tumor extent in a desmoplastic gastrointestinal cancer may not have the same clinical impact in a tumor already well characterized by MRI, FDG PET, or disease-specific tracers. The key clinical question is not whether FAPI PET detects more lesions in general, but whether additional findings are true, actionable, and likely to change treatment in a way that improves outcomes. Key scenarios and caveats are summarized in **Table 2** (33–41).

**Table 2:** Clinical scenarios in which FAP-targeted PET may add value and key interpretation caveats.

Clinical context	Potential added value	Main reporting caveat
Gastric cancer and peritoneal disease (33, 34)	Low background and desmoplastic stroma may improve detection of small deposits.	Postoperative change, adhesions, and benign repair can be FAP avid.
Pancreatic ductal adenocarcinoma (29, 39, 43, 44)	Helps evaluate stromal-rich primary or metastatic disease.	Pancreatitis and post-treatment fibrosis may mimic viable tumor.
HCC and biliary tract cancer (35–37)	May help when FDG is limited and BTC is desmoplastic.	Cirrhosis, cholangitis, and liver fibrosis require careful correlation.
Breast, lung, sarcoma, and glioblastoma (9, 13, 40, 41)	Useful for problem solving and whole-body target-expression assessment.	Evidence and uptake vary by histology, subtype, and prior treatment.
Benign fibro-inflammatory uptake (29, 43, 44)	Clarifies that FAP signal reflects activated repair biology, not malignancy alone.	Report as indeterminate when morphology or clinical context is not supportive.
Response and therapy selection (12, 42, 45–52)	Maps FAP-avid burden, heterogeneity, and discordant nonavid disease.	SUVmax alone is insufficient; standard imaging remains necessary.

### 4.3 Heterogeneity, prognosis, and response assessment

FAPI PET can show inter-lesion and intra-lesion heterogeneity. This may identify FAP-rich invasive margins, stromal-dominant metastases, or lesions that are biologically discordant from the rest of the disease. SUVmax alone is a poor summary because it ignores total FAP-avid burden, lesion volume, heterogeneity, and discordant nonavid disease. Volumetric metrics and lesion-level reporting may be useful for trials, but standardization of acquisition, reconstruction, reporting, and quantitative interpretation remains essential (12, 42).

The prognostic meaning of FAPI uptake remains under investigation. High FAP expression may capture biologically aggressive stromal programs including angiogenesis and immunoregulation, but prognostic claims should be disease-specific and adjusted for tumor burden, stage, treatment, and histology (14). At present, FAPI PET should be considered a candidate imaging biomarker rather than a validated pan-cancer prognostic test.

Response assessment is particularly complex after FAP-directed therapy. A fall in uptake may indicate tumor response, reduced fibroblast activation, treatment-related stromal depletion, or loss of target expression; increased uptake may reflect progression, wound healing, fibrosis, radiation injury, or immune-related fibro-inflammatory activation. After any FAP-targeted therapy, FAPI PET may be useful for documenting residual target-positive stroma, but it should not be used alone as a surrogate for viable tumor volume. CT, MRI, FDG PET or tumor-specific PET, biomarkers, symptoms, and histology when available remain necessary outcome measures (12, 29, 43, 44).

### 4.4 Pitfalls and benign fibro-inflammatory uptake

A necessary correction to early enthusiasm is that FAPI uptake is not synonymous with malignancy. Activated fibroblasts occur in healing wounds, arthritis, atherosclerosis, lung fibrosis, liver fibrosis, myocarditis, pancreatitis, post-surgical change, radiation injury, and other fibro-inflammatory or repair states (29, 43, 44). The term fibro-inflammatory is used here to emphasize that inflammation can stimulate fibroblast, myofibroblast, pericyte-like, or vascular smooth-muscle-cell-related FAP expression; it does not imply that FAP is primarily produced by immune cells.

A practical report should state the tracer, injected activity, uptake time, field of view, recent interventions, and relevant comparison imaging. Lesions should be described by location, intensity, CT or MRI correlate, and whether uptake is typical or atypical for malignancy. For potential RLT, the report should also document discordant nonavid lesions and normal-organ uptake because these features directly affect patient selection (12, 42–44).

This caution is especially important after surgery, biopsy, radiotherapy, infection, ablation, or systemic therapy. Reports should avoid categorical language when the CT or MRI correlate is absent or when benign explanations are plausible. Terms such as “FAP-avid lesion requiring correlation” or “indeterminate fibro-inflammatory uptake” are more appropriate than “metastasis” unless supported by the broader clinical context (43, 44).

## 5 FAP-targeted radionuclide therapy

### 5.1 Dosimetry and patient selection

FAP-targeted radionuclide therapy is biologically plausible and clinically feasible in selected patients, but it remains investigational. Patient selection should begin with FAP PET, yet SUVmax alone is insufficient. Clinicians need to know whether most clinically relevant lesions are FAP avid, whether uptake persists long enough to deliver dose, whether critical organs show uptake, and whether FAP-negative lesions could drive early progression (45–52).

Dosimetry is central to safe translation. Diagnostic FAP PET can screen for target expression, but therapy planning requires serial imaging or validated pharmacokinetic models to estimate time-integrated activity. Without these data, early uptake may overestimate deliverable absorbed dose. Dose constraints will differ by radioligand, radionuclide, prior therapy, marrow reserve, renal function, liver function, and disease distribution (45, 47–52). A further conceptual issue is whether FAP load predicts response. The intuitive assumption is reasonable: more target should permit more radioligand binding. However, response also depends on FAP molecules per cell, stromal fraction, spatial distance from tumor cells, perfusion, ligand penetration through dense matrix, internalization/retention, radionuclide range, lesion size, and radiosensitivity. Future trials should therefore relate quantitative PET metrics and serial dosimetry to absorbed dose and outcome rather than using baseline SUVmax as the sole eligibility gate (45, 47, 50, 51).

### 5.2 Clinical evidence to date

Clinical FAP-RLT evidence includes compassionate-use reports, small clinical series, dose-escalation studies, and prospective trials. In a prospective 90Y-FAPI-46 study, 119 patients were screened and 21 were treated across sarcoma and other cancers. Eligibility required progressive metastatic disease after approved options and high FAP expression, defined as SUVmax of at least 10 in more than 50% of tumors. Disease control by RECIST was reported in 8 of 21 patients (38%), including one partial response and seven stable diseases; grade 3/4 treatment-related adverse events occurred in 8 patients (38%), mainly thrombocytopenia and anemia. Reported organ dosimetry was relatively low for red marrow (about 0.04 Gy/GBq) and higher for kidney (about 0.53 Gy/GBq), illustrating feasibility but also marrow toxicity concerns in heavily pretreated populations (45). An earlier 90Y-FAPI-46 case series similarly supported feasibility but emphasized the need for prospective dosimetry and toxicity follow-up (46).

177Lu-FAP-2286 provides a clinically important example of a longer-retention ligand. In an early clinical study of 11 patients with advanced adenocarcinomas, 177Lu-FAP-2286 showed long tumor retention up to 10 days, mean administered activity of 5.8 GBq, mean kidney dose of about 1.0 Gy/GBq, red-marrow dose of about 0.05 Gy/GBq, and higher absorbed doses to some bone metastases. No grade 4 adverse events were reported; grade 3 events occurred in three patients, and most patients had progressive disease by RECIST at 6 to 8 weeks, with pain responses in selected cases (47). In the LuMIERE phase 1/2 program, dose escalation of 177Lu-FAP-2286 identified a recommended phase 2 dose of 9.25 GBq, with reported dose-limiting lymphopenia and hemoptysis and preliminary evidence of partial response or durable stable disease in a subset (48, 49).

Albumin-binding strategies have moved into clinical testing through 177Lu-LNC1004. In a first-in-human dose-escalation study of metastatic radioiodine-refractory thyroid cancer, 12 patients received cycles every 6 weeks at 2.22, 3.33, or 4.99 GBq; 3.33 GBq was selected as the recommended activity, with grade 3/4 hematotoxicity at higher activity and a reported overall response rate of 25% with disease control in 83% (50). In a subsequent single-center phase II study of 28 heavily pretreated metastatic solid tumor patients, 177Lu-LNC1004 at 3.33 GBq every 6 weeks produced disease control in 13 of 28 patients (46%), including four partial responses; the mean tumor absorbed dose was 4.69 ± 3.83 Gy/GBq, and grade 3/4 hematotoxicity occurred in six patients (21%), without grade 3/4 hepatotoxicity or nephrotoxicity (51).

Other emerging approaches include fractionated alpha-emitter therapy, such as 213Bi-FAPI-46 pilot experience, and alpha-emitter preclinical development with 225Ac-FAPI-46. These strategies are attractive for micrometastatic or heterogeneous disease but require careful attention to short-range dose distribution, renal and marrow toxicity, and the lack of mature comparative clinical data (25, 52). A detailed clinical evidence summary is provided in **Table 3**.

**Table 3:** Published clinical FAP-targeted radioligand therapy studies and limitations.

Agent/radionuclide	Population/setting	Key findings	Main limitations
90Y-FAPI-46 (45, 46)	Advanced sarcoma and other solid tumors; 21 treated after 119 screened in the dose-escalation experience.	Feasibility, palliative benefit, and disease stabilization in selected patients.	High screening attrition, heterogeneous uptake, and need for marrow/organ dosimetry.
177Lu-FAP-2286/LuMIERE (47–49)	Advanced FAP-expressing solid tumors in phase 1/2 development.	Early safety, dosimetry, and response signals support prospective testing.	Optimal cycle number, activity, and selection thresholds remain unsettled.
177Lu-EB-FAPI (20, 50)	Metastatic radioiodine-refractory thyroid cancer.	Albumin-binding approach increased exposure and showed first-in-human feasibility.	Longer circulation requires hematologic and whole-body dose monitoring.
177Lu-LNC1004 (51)	End-stage metastatic cancers in a single-center phase II study.	Reported antitumor activity with manageable toxicity in selected patients.	Single-arm design and heterogeneous tumor types limit generalization.
213Bi-FAPI-46 and 225Ac-FAPI-46 (25, 26, 52)	Pilot alpha-emitter use and preclinical alpha-emitter development.	High-LET strategy is attractive for small or heterogeneous lesions.	Sparse clinical data; renal, marrow, and microdosimetry questions remain.

### 5.3 Combination strategies and resistance mechanisms

FAP-RLT may be most useful in rational combinations rather than as a stand-alone replacement for systemic therapy. External-beam radiotherapy and DNA-damage response inhibitors may be explored in selected protocols for spatial dose intensification or radiosensitization, but these combinations require dedicated preclinical and clinical validation. Marrow reserve and normal-tissue repair must be protected because FAP uptake may occur in fibro-inflammatory tissue (45, 53, 54).

Immunotherapy combinations are attractive because stromal irradiation may alter antigen release, matrix barriers, cytokine gradients, and immune infiltration. Preclinical work with 177Lu-LNC1004 showed mechanistic support for combining FAP-targeted RLT with immune checkpoint blockade, including treatment-induced changes in the immune microenvironment (53). Similarly, 177Lu-FAP-2287 combined with PD-1 blockade enhanced antitumor activity in an immunocompetent FAP-expressing model, supporting clinical testing of 177Lu-FAP-2286 plus immune checkpoint inhibition (54). These data are hypothesis-generating; clinical protocols should prospectively monitor immune-related toxicity and distinguish immune-related fibro-inflammatory uptake from progression.

Resistance mechanisms can be grouped into target, delivery, and disease-level factors. Target resistance includes loss of FAP-positive stromal populations, emergence of FAP-negative CAF states, therapy-induced stromal remodeling, and discordance between tumor lesions. Delivery resistance includes inadequate penetration into dense matrix, heterogeneous perfusion, rapid washout, or subtherapeutic absorbed dose because of organ constraints. Disease-level resistance includes progression in FAP-negative tumor-cell-dominant lesions or sites outside the effective particle range. Repeat imaging may help identify target loss or new nonavid disease, but decisions to continue, switch, or stop treatment should be based on composite assessment rather than FAPI PET alone (45, 47–54).

## 6 Practical framework for clinical translation

### 6.1 Define the clinical question before imaging

FAPI PET should be requested for a specific decision point. Examples include whether a gastric cancer has occult peritoneal spread, whether FAPI-defined stromal extension could inform radiotherapy planning in a prospective protocol, whether a low-FDG-avid recurrence is present, whether a liver or biliary tract cancer is better characterized by stromal imaging, or whether a patient has sufficient whole-body target expression for a therapy trial. The report should answer that question directly and state how the result compares with standard imaging (33–37, 39, 45–52).

### 6.2 Minimum reporting elements and benign uptake checkpoints

For diagnostic imaging, minimum reporting elements include tracer identity, radiolabel, administered activity, uptake time, acquisition field, reconstruction protocol when relevant to quantification, lesion intensity, and comparison imaging (12). For therapy selection, reporting should also include the estimated percentage of FAP-avid disease burden, representative lesion uptake, discordant nonavid disease, normal-organ uptake, and any evidence of benign fibro-inflammatory uptake.

A practical checkpoint approach can reduce overinterpretation. Before labeling uptake as malignant, the reader should ask: Is there a CT or MRI correlate? Is the distribution typical for metastasis or local recurrence? Was there recent surgery, biopsy, ablation, radiotherapy, infection, pancreatitis, hepatitis, fracture, arthritis, or wound healing? Is FDG discordance biologically plausible? Would the finding change management enough to justify biopsy or follow-up? This conceptual layer goes beyond technical guidelines by explicitly linking interpretation to clinical action and risk of false-positive stromal uptake (12, 29, 43, 44).

### 6.3 Therapy selection and adaptive management

Reports intended to support therapy trials should identify dominant disease sites, follow-up target lesions, lesions discordant across imaging modalities, and organs that could become dose-limiting. Eligibility should incorporate lesion-level FAP avidity, tumor retention on delayed or serial imaging when available, marrow reserve, kidney function, liver function, prior radiotherapy, prior myelotoxic chemotherapy, and the presence of rapidly progressive FAP-negative disease. These elements are summarized in **Table 4** (12, 45–52).

**Table 4:** Therapy-oriented variables for FAP-targeted radiotheranostics.

Domain	Recommended assessment	Reason for importance
Target coverage	Baseline FAP PET with lesion-level uptake and estimate of FAP-avid disease burden (12, 45–52).	Confirms whether most clinically relevant disease is targetable.
Retention and dosimetry	Delayed imaging or serial quantitative imaging when therapy is considered (45–52).	Early high contrast does not necessarily mean adequate absorbed dose.
Normal-organ risk	Assess marrow, kidney, liver, salivary glands, and blood pool according to tracer and isotope (19, 45–52).	Defines safe activity, cycle interval, and stopping rules.
Prior treatment and reserve	Review chemotherapy, radiation, surgery, renal function, liver function, and marrow reserve (45, 47, 50, 51).	Predicts toxicity risk and helps interpret benign uptake.
Response assessment	Combine CT/MRI, FDG or disease-specific imaging, biomarkers, symptoms, labs, and FAPI PET (12, 43–52).	FAPI changes can reflect stromal remodeling rather than tumor-cell kill.
Adaptive management	Reassess nonavid progression, rapid washout, target loss, and resistant lesions (45–52).	Supports continuation, combination, or switching therapy.

Adaptive management should be prespecified. After one or two cycles, clinicians should review toxicity, laboratory reserve, symptoms, structural imaging, and molecular imaging. A fall in FAP uptake with stable or improved CT findings may support continued therapy; a fall in uptake with enlarging disease may reflect target loss; new FDG-positive/FAP-negative lesions may indicate escape; and persistent high uptake with acceptable organ dosimetry may support additional cycles. These rules need validation, but stating them prospectively will make future studies interpretable (45–52).

### 6.4 Trial design priorities and quality assurance

Diagnostic trials should measure clinical impact, not only lesion counts. Useful endpoints include changes in stage, treatment intent, surgical eligibility, radiation target volume, biopsy site selection, confidence in excluding metastatic disease, and downstream patient outcomes. Reference standards should be appropriate for each setting and may include pathology, follow-up imaging, intraoperative findings, or multidisciplinary adjudication (32–41).

Therapy trials should use strict eligibility criteria and predefined dosimetry where possible. Response criteria should combine structural imaging, molecular imaging, symptoms, quality of life, and laboratory toxicity. Control groups are challenging in rare or heavily pretreated populations, but randomized or matched designs will be needed if FAP-targeted therapy is to become routine (45–52).

Clinical implementation should involve nuclear medicine, radiology, medical oncology, radiation oncology, surgery, medical physics, radiopharmacy, and nursing. Local audit data should record indication, tracer, uptake time, key findings, management impact, follow-up confirmation, false-positive explanations, organ dosimetry where relevant, treatment cycles, and adverse events. Longitudinal studies should keep tracer, uptake time, scanner family, reconstruction parameters, and lesion-measurement methods as consistent as possible (12, 45–52).

## 7 Emerging trials and future directions

Future research should move from broad enthusiasm to disease-specific evidence. The strongest near-term indications are likely to involve tumors with prominent desmoplasia, low or variable FDG avidity, difficult peritoneal or local extension, liver or biliary tract settings where FDG is limited, or a realistic path to FAP-targeted therapy. Comparative trials should test whether FAPI PET improves decisions and outcomes beyond existing combinations of CT, MRI, FDG PET, endoscopy, surgery, and pathology (33–39, 45–52).

Representative ongoing or recently registered trials include diagnostic studies of 68Ga-FAPI-46 in resectable or borderline-resectable pancreatic cancer, 18F-FAPI-74 multicenter cancer detection studies, gastric cancer staging studies with FAPI PET/CT and laparoscopy, and therapeutic studies of 177Lu-FAP-2286 and other 177Lu-labeled FAP agents (49, 55–61). Trial status changes rapidly, and registry information should be checked at submission; however, these studies illustrate a transition from exploratory imaging toward disease-specific management-impact and dosimetry-driven therapy trials. Examples are summarized in **Table 5**.

**Table 5:** Representative ongoing or recently registered FAPI clinical trials.

Focus	Example trial(s)	Purpose and caveat
Pancreatic cancer imaging	68Ga-FAPI-46 in resectable or borderline-resectable PDAC, NCT05262855 (55).	Tests local/peritoneal staging and management impact.
Multicenter 18F-FAPI imaging	18F-FAPI-74 cancer detection studies, NCT06503146 and NCT05641896 (56, 57).	Supports distribution, harmonization, and disease-specific validation.
Gastric cancer staging	18F-FAPI PET/CT with laparoscopy, NCT07018661; Ga-68 FAPI PET/CT, NCT05898854 (58, 61).	Evaluates peritoneal disease and surgical triage; false-positive repair remains a caveat.
FAP-targeted radionuclide therapy	LuMIERE: 177Lu-FAP-2286, NCT04939610 (49).	Assesses safety, dosimetry, cycles, and response in FAP-expressing solid tumors.
Other 177Lu-FAP therapy	177Lu-JH04 in FAP-positive tumors, NCT06636617 (59).	May refine activity, toxicity, and patient-selection criteria.
Multimodality assessment	FAP PET/CT and PET/MR in malignant tumors, NCT06270394 (60).	Links diagnosis, staging, and treatment evaluation; protocols require standardization.

For therapy, the central challenge is to convert imaging contrast into safe absorbed dose. Progress will require ligands with better tumor residence time, serial-imaging dosimetry, organ-specific toxicity thresholds, rational radionuclide choice, and eligibility criteria that integrate FAP-avid burden with nonavid disease. The field also needs consensus on what level of FAP expression is sufficient for therapy and how discordant disease should be handled (15–26, 45–52).

Biological integration will be equally important. FAPI PET should be correlated with histopathologic FAP expression, CAF phenotypes, ECM signatures, immune infiltration, perfusion, hypoxia, and genomic features. Such studies may explain why some lesions are strongly FAPI avid, why others are not, and which combinations are most likely to improve clinical outcomes (4, 5, 14, 53, 54).

A further priority is harmonization. Multicenter studies need comparable acquisition times, reconstruction parameters, injected activity documentation, lesion selection rules, and definitions of FAP-avid disease burden. Harmonization does not mean that every tracer must be used identically; rather, each tracer should have a clear evidence-based protocol so that results can be compared across institutions and incorporated into meta-analyses, guidelines, and regulatory dossiers (12, 27–32).

## 8 Discussion and conclusion

FAPI-based radiotheranostics has changed how nuclear oncology can interrogate the TME. Its clinical value lies in imaging activated stroma across the whole body, providing information that differs from glucose metabolism and conventional morphology. The method is most useful when it is tied to a concrete clinical question and interpreted with awareness of benign fibro-inflammatory uptake (1–14, 29, 43, 44).

The therapeutic component is less mature than the imaging component. Current data support feasibility, biologic rationale, and selected clinical responses, but routine adoption requires better ligands, prospective dosimetry, standardized eligibility criteria, and evidence of benefit in defined patient groups. The most important safety signals to monitor are not only generic radiation risks but also FAP-RLT-specific issues: marrow suppression from prolonged blood-pool activity or prior therapy, renal and bowel dose depending on ligand handling, uptake in wound healing or inflamed tissue, and possible effects on normal tissue repair (45–52).

For oncology clinicians and researchers, the key message is pragmatic. FAPI PET is a promising method for mapping activated stroma, but it should not be interpreted in isolation from established oncologic imaging. The technology is most persuasive when it solves a defined problem: staging a tumor with low FDG avidity, clarifying equivocal peritoneal disease, evaluating HCC or biliary tract cancer in which FDG has limitations, selecting a biopsy target, documenting whole-body target expression, or supporting a protocolized therapy study. These narrower, testable roles are more likely to lead to durable clinical adoption than broad claims of pan-cancer superiority (27–41, 45–52).

A realistic translation pathway is therefore a closed-loop model: standardized FAP PET for detection and target-expression assessment, explicit benign uptake checkpoints, delayed imaging or dosimetry for therapy selection, agent-specific radionuclide choice, toxicity monitoring, and adaptive reassessment using standard oncologic outcome measures as well as molecular imaging. If these steps are implemented prospectively, FAPI radiotheranostics could become a useful part of precision oncology without overstating what the current evidence can support (12, 45–54).
